# Diffuse Nocardial Spinal Subdural Empyema: Diagnostic Dilemma and Treatment Options

**DOI:** 10.7759/cureus.1795

**Published:** 2017-10-24

**Authors:** Zaid Aljuboori, Mayur Sharma, Thomas Altstadt

**Affiliations:** 1 Department of Neurosurgery, University of Louisville School of Medicine

**Keywords:** nocardiosis, subdural empyema, brain abscess, cns infection

## Abstract

Nocardiosis of the central nervous system and spine, in particular, is a rare infection with significant morbidity and mortality. Treatment is usually with antibiotics and surgical drainage or biopsy. The authors report a case of a 49-year-old man who presented with chronic lower back pain and paraplegia. He was found to have spinal subdural empyema caused by Nocardia farcinica. Laminectomy and sampling of the subdural collection were performed, and the patient was treated with triple intravenous antibiotics (linezolid, amikacin and ciprofloxacin). There was no neurological recovery at follow-up. Spinal nocardiosis should be considered in the differential diagnosis of immunocompromised patients who present with diffuse spinal epidural/subdural or spinal cord abscesses, appropriately unresponsive to antibiotics. Our case provides an insight into the management challenges of this rare disease.

## Introduction

The Nocardia species are aerobic, Gram-variable, rod-shaped bacteria. Nocardiosis is considered as an opportunistic infection that usually affects immunocompromised patients [1].

Nocardiosis is a rare infection, with about 500-1000 new cases reported each year in the United States. It usually affects patients with cell-mediated immunodeficiency, although 42% of patients have no known immunodeficiency [1]. Infection is acquired primarily by inhalation from the environment, the lung being the primary site of involvement [2]. Extra-pulmonary involvement occurs through hematogenous dissemination or contiguous spread [3-4]. The central nervous system (CNS) is the most common extra-pulmonary site and can be considered as the primary site of nocardiosis [4]. CNS involvement occurs in about 44% of disseminated cases [1,5], and it accounts for 2% of all cerebral abscesses [2,6]. More than half the patients with a CNS disease are immunocompromised [2,7]. CNS involvement can also occur without evidence of systemic infection [1]. Nocardia generally produces multifocal brain abscesses, but occasionally it can manifest as meningitis, spinal cord abscess, spinal epidural abscess, or vertebral osteomyelitis [8]. Mortality from CNS nocardiosis can reach up to 55% [1,8]. Spinal nocardiosis is rare, with few reported cases in the literature [4-5].

We report the first case of diffuse spinal subdural empyema caused by Nocardia farcinica in a patient with a history of a treated nocardial brain abscess. Our patient is an immunocompetent male with a history of intravenous drug abuse and chronic hepatitis C virus infection. He presented with lower back pain and paraplegia for one month. The disease was controlled with antibiotics but no neurological recovery was achieved.

## Case presentation

Our patient is a 49-year-old Caucasian male with a history of intravenous (IV) drug abuse, chronic hepatitis C virus infection, smoking and chronic obstructive airway disease. He had a past history of nocardial brain abscess that was treated with aspiration and antibiotics, one year prior to current presentation. Six weeks prior to admission, the patient underwent lumbar epidural steroid injections for lumbar radiculopathy and chronic low back pain. The patient subsequently started to develop bladder/bowel incontinence and progressive bilateral lower extremity weakness. He presented in the emergency room (ER) with bilateral lower extremity weakness and sphincterian incontinence.

**Figure 1:**
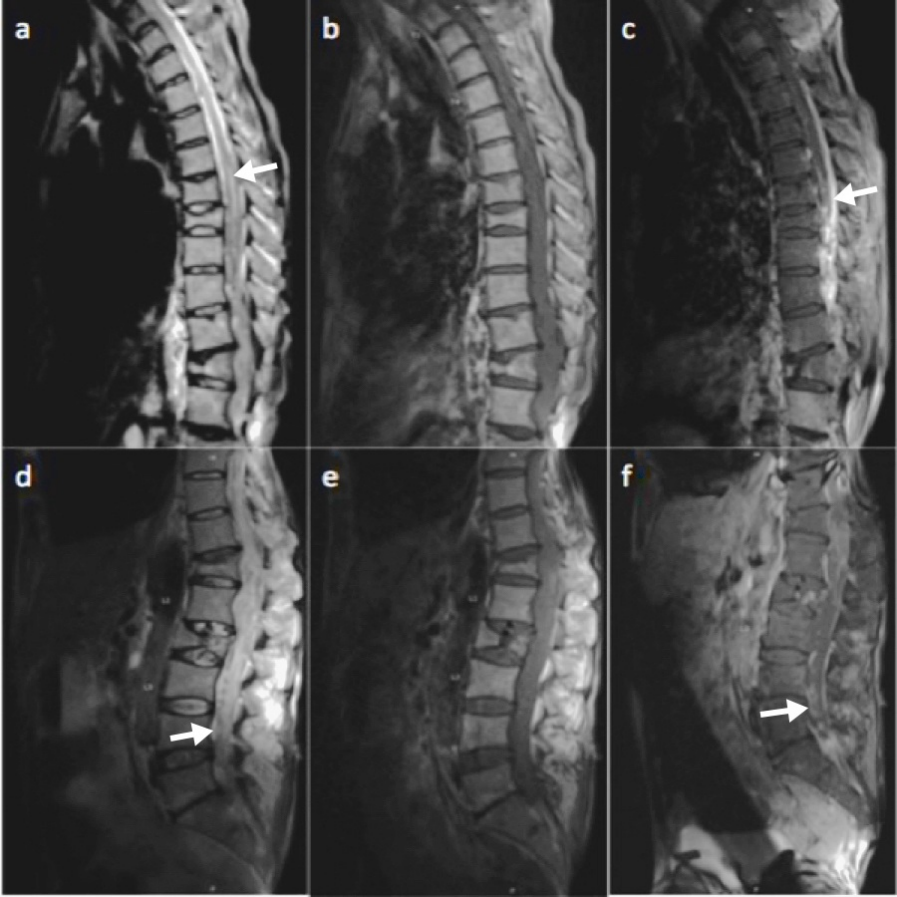
MRI scan of thoracic (upper row) and lumbar spine (lower row) with and without contrast [a] T2W shows a diffuse hyperintensity (arrow) involving the central portion of the thoracic spinal cord. [b] T1W C- shows a diffuse low signal involving the thoracic spinal cord. [c] T1W C+ shows a diffuse dural and pial enhancement (arrow) with no definitive focal fluid collections. [d] T2W shows a heterogeneous signal throughout the lumbar spinal canal (arrow) with areas of multiloculated T2 hyperintensity. [e] T1W C- shows a diffuse low signal involving the cauda equina. [f] T1W C+ shows a multiloculated enhancement of the cauda equina (arrow) and the peripheral margins of the lumbar spinal canal. MRI: magnetic resonance imaging

On clinical examination, the patient had paraplegia, T12 sensory level, no rectal tone, saddle paresthesia, with full strength of upper extremities. A magnetic resonance imaging (MRI) scan of the neuroaxis revealed extensive heterogeneously enhancing multifocal fluid collections within the thoracic and lumbar spine pleading for hemorrhage versus epidural abscess (Figure 1), no brain involvement was identified on a cerebral MRI scan. Human immunodeficiency virus (HIV) screening was non-reactive and blood cultures were negative on two occasions. An interventional radiology-guided tissue biopsy was unsuccessful due to a lack of organized fluid. He was started on empiric intravenous antibiotics (vancomycin, trimethoprim and sulfamethoxazole (TMP/SMX)) due to his history of nocardial brain abscess.

Therefore, a decision was made to obtain an open tissue biopsy for a microbiology and pathology examination. An L4-L5 lumbar laminectomy was performed; intra-operatively, there was no evidence of epidural fluid collection. The dura showed inflammatory changes with thrombosed epidural veins. The dura was opened and a thick yellowish purulent material was expressed from the subdural space.

A microbiological examination of the subdural fluid revealed modified, acid-fast, Gram-positive branching rods suggestive of Nocardia farcinica (susceptible to amikacin, ciprofloxacin, linezolid and moxifloxacin). The patient was initially treated empirically with IV meropenem/IV amikacin/IV TMP/SMX and later changed to amikacin/ciprofloxacin and linezolid according to the final antibiotic sensitivity results.

The patient reported gradual improvement in his low back pain, with, however no positive outcome in his neurological status. He was discharged to a long-term rehabilitation facility on a six weeks' course of IV antibiotics (amikacin, ciprofloxacin and linezolid) followed by oral bactrim and ciprofloxacin for one year. 

**Figure 2:**
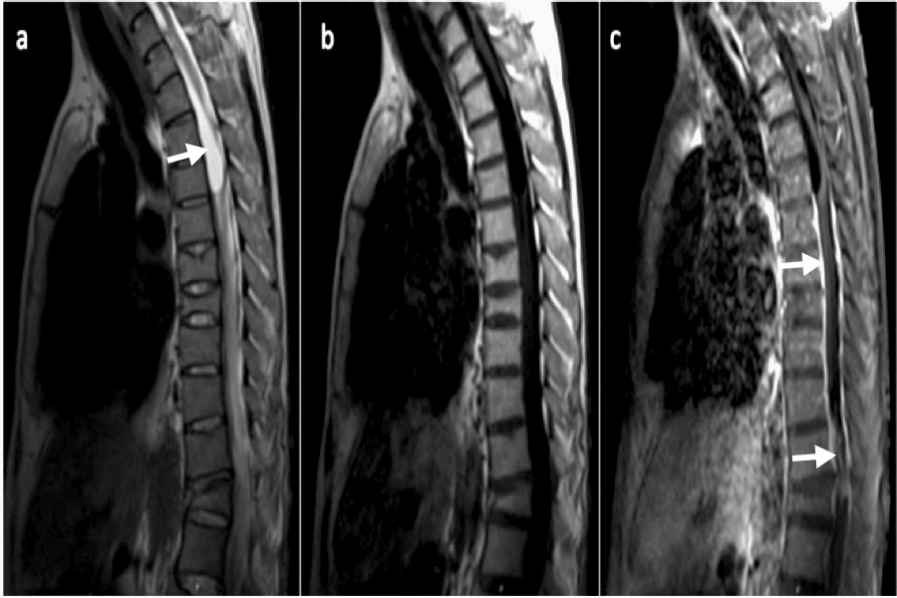
MRI of thoracic spine with and without contrast [a] Sagittal T2W shows an intradural and partially intramedullary elongated cystic structure (arrow) in the upper thoracic spine with marked distortion and compression of the spinal cord. Also, the spinal cord with abnormal T2 hyperintensity is seen with septated and tethered components diffusely. [b] T1W C- and [c] T1W C+ show an epidural and intradural enhancement with intramedullary irregular areas of enhancement in the distal cord (arrows). MRI: magnetic resonance imaging

The patient was readmitted to our hospital four months following discharge with an E. coli urinary tract infection and an infected stage IV sacral decubitus ulcer due to methicillin-resistant Staphylococcus aureus. Further inquiry revealed that he was not compliant with the oral antibiotic treatment and was also lost to follow-up. An MRI of the thoracolumbar spine (with gadolinium contrast, Figure 2) revealed minimal improvement in the degree of epidural and intradural enhancement and a minimal decrease in the intramedullary irregular areas of enhancement in the distal spinal cord. Distortion of the spinal cord with abnormal T2 hyperintensity was present with diffuse septated and tethered components. He was put back on IV antibiotics (amikacin, ciprofloxacin and linezolid) and discharged to a nursing home. 

## Discussion

Nocardia is a Gram-positive, partially acid-fast, catalase negative and urease positive bacterium that grows aerobically [2,4]. It was first identified by Edmond Nocard in 1889 [1]. Nocardia species are facultative intracellular pathogens that interfere with oxidative inhibiting processes and the fusion of phagosomes and lysosomes and prevent phagosomal acidification after being taken up by phagocytes [1,6-7]. The organism can pass through endothelial walls to invade the brain where it infects both microglia and astrocytes [7].

A Nocardia CNS infection typically presents as a cerebral abscess, but it can also present as meningitis, diffuse cerebral infiltration without focal lesion or granuloma with giant cells [7]. On the other hand, the involvement of the spinal cord is very rare; only a few cases have been reported [4-5]. The lesions are singular in 54%, multiple in 38% and of an unknown number in 8% of patients. The principal presenting symptoms are a focal deficit in 42%, non-focal findings in 28% and seizures in 30% of the patients [6]. Our patient presented with lower back pain and paraplegia for a month following lumbar epidural steroid injections for lumbar radiculopathy. Since our patient had a history of treated cerebral nocardiosis, his spinal involvement was likely secondary to the activation of the latent nocardial infection. This fact should be taken into consideration when treating patients with a history of nocardiosis.

The optimal management of CNS nocardiosis remains unclear due to the rarity of the infection and a lack of prospective controlled trials [1-2]. High-dose sulfonamides or the TMP/SMX combination is the therapy of choice for Nocardia [1-2,4-5, 8]. Alternative antimicrobial agents, such as carbapenems, cephalosporins, aminoglycosides, quinolones, macrolides or tetracyclines, can be used in patients who cannot tolerate sulfa drugs or for resistant microorganisms [6]. The treatment regimen recommended for patients with CNS nocardiosis include parenteral trimethoprim-sulfamethoxazole (TMP/SMX, 10-20 mg/kg/d in three, divided doses over 24 hours) with imipenem 500 mg qid and amikacin 15 mg/kg/d if other organs are involved for at least six weeks [9]. Certain Nocardia species are resistant to amikacin; therefore, Linezolid can be used instead [1]. If the patient shows clinical and radiological improvement with the intravenous regimen, treatment can be converted to two-drug oral therapy [9]. Although the optimum duration of antibiotic therapy is uncertain, long-term suppressive therapy is strongly recommended. Immunocompetent patients with CNS nocardiosis should be treated for 12 months [10].

Treatment of nocardial spinal abscesses depends on the size, location and appearance of the abscess. Nocardial spinal epidural abscesses can be treated with either open drainage through laminectomy or with percutaneous drainage [5], However, in cases of spinal cord abscess, there are no clear recommendations. Based on two reported cases [4-5], it appeared that laminectomy is usually performed to aspirate or enucleate the abscess. In our case, it was not possible to drain the entire subdural empyema because of the extensive involvement of both thoracic and lumbar spine as well as the consistency of the subdural collection. Therefore, open biopsy for culture and sensitivity was considered. Despite advances in imaging techniques, surgical procedures and antibiotics, the morbidity and mortality associated with CNS nocardiosis are very high [1,8].

## Conclusions

Diffuse spinal nocardiosis is a rare infectious disease with high morbidity and mortality. A nocardial infection should be considered in immunocompromised patients presenting with spinal epidural/subdural or spinal cord abscesses, especially if they fail to respond appropriately to antibiotics. Early evaluation with MRI scans and biopsy/drainage of the abscess may provide an early and accurate diagnosis. A multidisciplinary approach, including antibiotics guided by sensitivity testing as well as surgical treatment when appropriate, can be considered for treating a nocardial infection.
